# Adverse events in psychiatry: a national cohort study in Sweden with a unique psychiatric trigger tool

**DOI:** 10.1186/s12888-020-2447-2

**Published:** 2020-02-04

**Authors:** Lena Nilsson, Madeleine Borgstedt-Risberg, Charlotta Brunner, Ullakarin Nyberg, Urban Nylén, Carina Ålenius, Hans Rutberg

**Affiliations:** 1 Department of Anaesthesiology and Intensive Care, Department of Biomedical and Clinical Sciences, Linköping University, Linköping, Sweden; 2 Department of Anaesthesiology and Intensive Care, University Hospital, 583 81 Linköping, Sweden; 3 Centre for Organisational Support and Development (CVU), Region Östergötland, Linköping University, Linköping, Sweden; 4 Department of Psychiatry, Kalmar County Council, Kalmar, Sweden; 5 Stockholm Centre for Psychiatric Research and Education, Department of Clinical Neuroscience, Karolinska Institutet, Stockholm, Sweden; 6 National Board of Health and Welfare, Stockholm, Sweden; 7 Swedish Association of Local Authorities and Regions, Stockholm, Sweden

**Keywords:** Adverse event, Patient harm, Patient safety, Trigger tool, Psychiatry

## Abstract

**Background:**

The vast majority of patient safety research has focused on somatic health care. Although specific adverse events (AEs) within psychiatric healthcare have been explored, the overall level and nature of AEs is sparsely investigated.

**Methods:**

Cohort study using a retrospective record review based on a two-step trigger tool methodology in the charts of randomly selected patients 18 years or older admitted to the psychiatric acute care departments in all Swedish regions from January 1 to June 30, 2017. Hospital care together with corresponding outpatient care were reviewed as a continuum, over a maximum of 3 months. The AEs were categorised according to type, severity and preventability.

**Results:**

In total, the medical records of 2552 patients were reviewed. Among the patients, 50.4% were women and 49.6% were men. The median (range) age was 44 (18–97) years for women and 44.5 (18–93) years for men.

In 438 of the reviewed records, 720 AEs were identified, corresponding to the AEs identified in 17.2% [95% confidence interval, 15.7–18.6] of the records. The majority of AEs resulted in less or moderate harm, and 46.2% were considered preventable. Prolonged disease progression and deliberate self-harm were the most common types of AEs. AEs were significantly more common in women (21.5%) than in men (12.7%) but showed no difference between age groups. Severe or catastrophic harm was found in 2.3% of the records, and the majority affected were women (61%). Triggers pointing at deficient quality of care were found in 78% of the records, with the absence of a treatment plan being the most common.

**Conclusions:**

AEs are common in psychiatric care. Aside from further patient safety work, systematic interventions are also warranted to improve the quality of psychiatric care.

## Background

In the last few decades, there has been a growing interest in patient safety worldwide, with the vast majority of research focusing on somatic healthcare. Although many patient safety risk factors in somatic settings also apply to psychiatric and mental health care, recognising specific adverse events (AEs) that are unique to mental healthcare is important. Examples of these AEs are those regarding seclusion and use of restraint, self-harm and suicidal behaviour. Some of these events have been investigated, such as suicide and self-harm [1–5], medication errors [6, 7] and seclusion and restraint [8, 9], but the overall level and nature of AEs within psychiatry have been sparsely explored [10–12].

The retrospective medical record review is an established and validated method to identify AEs in healthcare [13–15]. A list of criteria (triggers) is commonly used to identify details in the records that often are associated with the presence of AEs. The trigger tool methodology gives information on the incidence, nature, preventability and consequences of AEs that can be used in systematic quality improvement work.

Most studies on the patient safety trigger tool have been undertaken in adult hospital somatic care. In Sweden, the trigger tool methodology has been used for somatic care at a national level since 2012 [16, 17].

The IHI Global Trigger Tool [18] has been used since 2008. Trigger tools have also been adapted for specific areas, such as paediatric care [19, 20], primary care [21] and home healthcare [22]. In mental health services there is a trigger tool for measuring adverse drug events [23], and a combined trigger tool for detecting somatic AEs and mental health-related patient safety incidents [12].

In Sweden patients with less severe mental disorders, primarily anxiety and depression, are primarily taken care of in the primary care system by a general practicioner (mental health services), while patients having more severe conditions are referred to psychiatric departments for hospital or outpatient care. In this study we focused on the latter adult patient population.

This study aimed 1) to develop a national trigger tool for adult psychiatric care and 2) to describe the incidence, nature, preventability and severity of AEs in adult psychiatric healthcare.

## Methods

### Development and implementation of the Swedish national psychiatric trigger tool

The IHI Global Trigger Tool [18] has been used in Swedish healthcare since 2008. Following a national patient safety initiative (2011–2014), all acute care hospitals have their own somatic review teams, and a national database was developed by the Swedish Association of Local Authorities and Regions.

In 2012, the representatives of psychiatric healthcare and the mental health patient organisations started developing a psychiatric trigger tool. Together, the risk areas specific to psychiatric hospital care were identified such as coercion, suicide risk assessment, medication, threats and violence, non-compliance to treatment plan, transitions in healthcare and the diagnostic process. An AE was defined as suffering, a physical or psychological unfavourable event or disease or death as a result of the contact between a patient and healthcare. An AE was categorised into one of 22 different types (Table 1)*.* Each AE could only be categorised into one specific type.

**Table 1:** Area and type of AEs

Area of AE	Type of AE	Examples
Mental injury	1. Suffering	Insecurity, fear after threat/violence, discomfort, stigmatisation
	2. Insult	In connection with coercive measures, sexual abuse
Prolonged disease progression	3. Untreated condition	Incomplete investigation with incorrect diagnosis Unwanted effect in psychotherapy Insufficient assessment during ongoing treatment
	4. Insufficient effect of treatment	
	5. Interrupted treatment	
	6. Disease worsening	
Deliberate self-harm	7. Suicide	
	8. Suicide attempt	
	9. Self-harm without suicidal intent	
Medication-related injury	10. Metabolic influence	Acute dystonia, dyskinesia, akathisia, affected renal function, confusion, sedation, hypotonia, malignant neuroleptic syndrome, serotonin syndrome, medication error
	11. Extrapyramidal symptoms	
	12. Allergic reaction	
	13. Drug addiction	
	14. Other drug-related harm	
Illegal/unethical treatment	15. Illegal restraint	Detained after administrative mishaps
	16. Measures without support in law	
Physical injury	17. Anaesthesia-related injury	Tooth damage, breathing and/or circulation failure, fracture, haemorrhage, infection, memory disorder after electroconvulsive therapy
	18. Falls	
	19. Pressure ulcer category 2–4	
	20. Cognitive failure	
	21. Other physical harm	
Others	22. Other AEs	

*AE* Adverse event

Much effort was put into developing guidelines on how to define an AE. A patient perspective was considered by reviewing hospital care and the corresponding outpatient care as a continuum for each patient. In contrast to somatic care in which only hospital care has been reviewed when the trigger tool review method is used, we assessed all documented care during a three-month period for each medical record.

Based on the identified risk areas and known types of AEs, triggers with the possibility of identifying harm were formulated. AEs were categorised into one of four severity categories: Less–discomfort or insignificant harm, Moderate–temporary disability, Significant–permanent disability and Catastrophic–permanent substantial disability, death.

An AE was categorised as preventable or not by using a graded scale of four options: 1) ‘not preventable’, 2) ‘probably not preventable’, 3) ‘probably preventable’ and 4) ‘certainly preventable’. AEs resulting from omission were regarded as preventable. The manual gave detailed instructions on the difficult assessment of preventability (Table 2)*.* AEs classified as categories 1 and 2 are denoted as non-preventable, and AEs labelled as 3 and 4 as preventable in the following text and tables.

**Table 2:** Example of preventability assessment instructions

T1 Coercion treatment–administrative failure	
Definition	Failure in the documentation of decisions according to LPT or LRV or Time limits given for coercion were not followed or documented correctly.
To remember	The violation of rules may lead to the patient being custodial without legal support or not having his/her rights catered for, e.g., information and the possibility to appeal against coercion decisions. Is the documentation of decisions adequate?
AE that can be found	The patient is exposed to authority handling without legal support, custodial and psychic suffering.
Preventability	If time limits were not followed in connection with coercion or if the documentation of coercion is incorrect, the AE is considered preventable.

AE: Adverse event; LPT, LRV: Swedish laws of coercion in psychiatric and forensic healthcare

The trigger tool underwent a pilot test of 471 records from 17 of the 21 regions in the country. The final version was completed in 2015. Divided into 5 modules, namely, treatment, drugs, coercive treatment, medicine and continuity and transition, 36 triggers were described (Table 3). Since 2015, centralised education has been offered regularly to new members in the ‘review teams’ in psychiatric healthcare.

**Table 3:** The final trigger tool

Treatment	
V1	Absence of a care plan
V2	Absence of an intervention plan
V3	Lack of suicide risk assessment
V4	Lack of review of crime relapse risks
V5	Falls
V6	Documentation of failure
V7	Consultation with a physician on call/doctor from another specialty
V8	Change in diagnosis
V9	Self-harm
V10	Undesired effect of treatment other than medication
V11	Threats, violence and inappropriate behaviour
V12	Increased surveillance
V13	Lack of documented physical observations
V14	Absence of family contact
V15	Others
Drugs	
Y1	Absence of the alcohol use disorder identification test (AUDIT)
Y2	Absence of the addiction severity index (ASI)
Y3	Absence of the expiratory alcohol test
Y4	Absence of a urinary lab test when addiction is suspected
Coercive treatment	
T1	Coercion treatment–administrative failure
T2	Coercion
T3	Conversion from voluntary treatment to coercion (emergency law)
T4	Police assistance
Medicine	
B1	Use of three or more different antipsychotic drugs
B2	Treatment with anticholinergics
B3	Use of more than five different psychotropic drugs
B4	More than three benzodiazepines or treatment for more than 6 months
B5	Faults in screening for metabolic risk factors during antipsychotic treatment
B6	Lack of regular tests for medication with lithium, methylphenidate, methadone/buprenorphine or clozapine
B7	Medication, others
Continuity and transition	
R1	Unplanned contact with a psychiatric acute unit
R2	Reinstatement within 30 days
R3	Change in treatment unit
R4	Unplanned discharge
R5	Lack of doctors’ visit during the last 12 months in outpatient care
R6	Lack of an accountable primary physician

### Inclusion criteria and sampling

Randomly selected records of patients aged 18 years or older who had been discharged from psychiatric hospital care between January 1, 2017 and June 30, 2017 were reviewed. Each record was assessed for a three-month period. Both hospital stays and associated outpatient care were included in the assessment, which was discontinued at a minimum of 30 days before the time for review to enable the identification of AEs not obvious until after discharge. All 21 regions in Sweden participated in the study.

### Review process

Each hospital had its own review team that consisted of one or two nurses or other mental healthcare professionals with long experience and at least one physician. All team members were senior level with special training in the record review method and an interest and knowledge in the field of patient safety.

The process started with a nurse or a mental healthcare professional screening the records for the presence of triggers and possible AEs. The full record including observational notes, medication charts, laboratory tests etc. was examined. The second review stage had a dual purpose: 1) The team assessed the occurrence of AEs, which were categorised according to type, severity and preventability. 2) The identification of triggers was used to assess quality of care, including level of compliance to local guidelines and clinical routines, regardless of the occurrence of an AE. Nineteen of the 36 triggers could be used for determining the lack of quality. Examples of such triggers were ‘absence of care plan’, ‘absence of family contact’, ‘lack of suicide risk assessment’ and ‘lack of documented physical observations’ in connection with inpatient care. There was no time limit for the review stages. No assessment of interrater reliability was performed.

### Statistics

Data are presented as the number (percent), median (range) or mean [95% confidence interval (CI)]*.* Comparison of the proportions was made using a chi-squared test. We calculated the CIs using a normal distribution approximation. A *p* value of less than 0.05 was considered significant. All statistical calculations were made using SPSS version 25 (IBM, New York, USA).

## Results

In total, 2552 medical records were reviewed. This number corresponded to 3% of the national psychiatric hospital care during a six-month period and was divided by the regions in proportion to the total number of hospital stays. The distribution between sexes was equal, with 50.4% women and 49.6% men. The median (range) age was 44 (18–97) years for women and 44.5 (18–93) years for men. A total of 707 (28%) of the records included hospital care only, and the rest covered hospital and outpatient care.

Out of the 2552 records reviewed, 438 (17.2%, 95% CI 15.7–18.6) records had a total of 720 identified AEs. Among the reviewed records, 8.0% (95% CI 7.0–9.1) had AEs that were classified as preventable. In total, 46,2% of the identified AEs were assessed as preventable. No significant difference was found in the incidence of AEs among the different age groups.

The AEs mostly resulted in less or moderate harm: less harm (discomfort, insignificant harm) accounted for 41.0%; moderate harm (transient disability) accounted for 46.1%, severe harm (permanent moderate disability) accounted for 12.5% and catastrophic harm (permanent major disability, death) accounted for 0.4%. Prolonged disease progression and deliberate self-harm were the most common types of AEs. The identified types of AEs differed between hospital and outpatient care (Table 4).

**Table 4:** AEs in psychiatric care

Area of AE	Total number of AEs n (%)	Preventable AEs n (%)	AEs in hospital care n (%)	AEs in outpatient care n (%)
Prolonged disease progression	214 (30)	148 (69)	102 (23)	112 (41)
Deliberate self-harm^a^	177 (25)	42 (24)	106 (24)	71 (26)
Mental injury	139 (19)	68 (49)	106 (24)	33 (12)
Medication-related injury	84 (12)	27 (32)	52 (12)	32 (12)
Physical injury	68 (9)	21 (31)	60 (13)	8 (3)
Other	33 (4)	24 (73)	18 (4)	15 (6)
Illegal/unethical treatment	5 (1)	3 (60)	5 (1)	0 (0)
Total	720 (100)	333 (46)	449 (100)	271 (100)

^a^Among the AEs in the area of *deliberate self-harm*, the AE type *suicide attempt* was significantly more common in outpatient care, and the incidence of deliberate self-harm did not differ between patients in hospital care and those in outpatient care

*AE* Adverse event

**Fig. 1:**
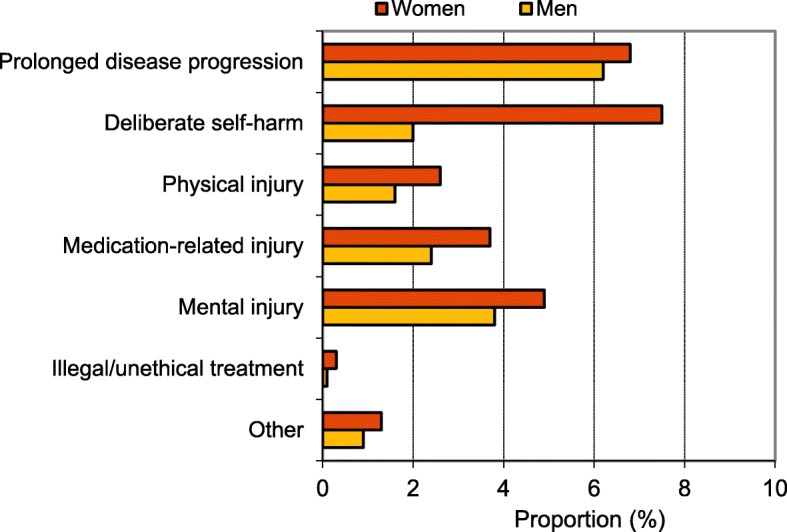
Proportion of care episodes with adverse events for women and men

AEs were significantly more common in women (21.5%) than in men (12.7%) (*p* < 0.001). A significant gender difference was also seen for preventable AEs at 9.7% for women and 6.3% for men (*p* = 0.002). The most prominent difference was found in the category deliberate self-harm, with an incidence of 7.5% in women and 2.0% in men (Fig. 1). A significant gender difference in the total material remained after the removal of this category.

A detailed description of the areas and types of AEs is shown in Table 5. ‘Prolonged disease progression’ was the most common AE area. Among the specific AE types, ‘disease worsening’ was the most prominent. From a patient perspective, coercive measures, such as the manual or mechanical restraint of the patient’s limbs or body to prevent free movement, are a violation. Thus, we regarded this measure as an AE. Such AEs may be preventable depending on the situation. This was assessed by the review teams.

**Table 5:** Incidence of AEs categorised according to area and type

Area and type of AE	Incidence (%)	
Mental injury	4.3	
Suffering		3.5
Insult		1.3
Prolonged disease progression	6.5	
Untreated condition		1.5
Insufficient effect of treatment		2.3
Interrupted treatment		0.9
Disease worsening		3.0
Deliberate self-harm	4.8	
Suicide		0.0
Suicide attempt		2.2
Self-harm without suicidal intent		3.1
Medication-related injury	3.1	
Metabolic influence		0.5
Extrapyramidal symptoms		0.6
Allergic reaction		0.2
Drug addiction		0.3
Other drug-related harm		1.6
Illegal/unethical treatment	0.2	
Illegal restraint		0.1
Measures without support in law		0.1
Physical injury	2.1	
Anaesthesia-related injury		0.0
Falls		1.1
Pressure ulcers category 2–4		0.0
Cognitive failure		0.2
Other physical harm		0.9
Other	1.1	

*AE* Adverse event

Triggers pointing at deficient quality of care were found in 1995 (78%, 95%CI 76.5–79.8%) of the records. The most common triggers were ‘absence of care plan’, ‘absence of family contact’, ‘lack of suicide risk assessment’ and ‘lack of documented physical observations’ related to inpatient care (Table 6). More than one type of trigger indicating deficient care quality was usually found during a single care episode.

**Table 6:** Incidence of care episodes with triggers pointing at deficient quality of care

Trigger	Number (Percent)
Treatment	
Absence of care plan	926 (36.3)
Lack of documented physical observations	582 (22.8)
Lack of suicide risk assessment	401 (15.7)
Absence of family contact	272 (10.7)
Absence of intervention plan	235 (9.2)
Lack of review of crime relapse risk	19 (0.7)
Drugs	
Absence of the alcohol use disorder identification test (AUDIT)	319 (12.5)
Absence of a urinary lab test when addiction is suspected	218 (8.5)
Absence of the expiratory alcohol test	107 (4.2)
Absence of the addiction severity index (ASIA)	50 (2.0)
Coercive treatment	
Coercion treatment–administrative failure	76 (3.0)
Medicine	
Use of more than five different psychotropic drugs	251 (10.1)
More than three benzodiazepines or treatment for more than 6 months	247 (9.7)
Faults in screening for metabolic risk factors during antipsychotic treatment	202 (7.9)
Lack of regular tests for medication with lithium, methylphenidate, methadone/buprenorphine or clozapine	187 (7.3)
Use of three or more different antipsychotic drugs	75 (2.9)
Continuity and transition	
Lack of an accountable physician	367 (14.4)
Unplanned discharge	268 (10.5)
Lack of physician’s visit during the last 12 months in outpatient care	69 (2.7)

The proportion of patients with less or moderate harm was 15.9% and severe or catastrophic harm 2.3%. The different areas of AEs, gender and the degree of preventability of the severe or catastrophic AEs are displayed in Table 7. Thirty-six (61%) of the patients with severe or catastrophic harm were women and 23 (39%) were men. The distribution in age less than or 45 years and older was equal (32 and 27 patients, respectively), as was the distribution between hospital and outpatient care (31 and 35 patients, respectively).

**Table 7:** Incidence of severe and catastrophic AEs categoridsed according to area, gender and preventability

Area of AE	Severe and catastrophic AEs *n* (%)	Men/women *n*	Preventable AE *n* (%)
Prolonged disease progression	44 (20.6)	18/26	38 (86.4)
Deliberate self-harm	18 (10.2)	8/10	10 (55.6)
Mental injury	12 (8.6)	3/9	11 (91.7)
Medication-related injury	11 (13.1)	4/7	5 (45.5)
Physical injury	2 (2.9)	0/2	0 (0)
Other	6 (18.2)	2/4	3 (50.0)
Illegal/unethical treatment	0 (0)	0/0	0 (0)
Total	93 (12.9)	35/58	67 (72.0)

*AE* Adverse event

## Discussion

To the best of our knowledge, this study is the first multicentric, national study to report on psychiatric AEs at a national level. AEs occurred in 17% of the investigated care episodes, mostly resulting in less or moderate harm and with almost half considered as preventable. Women were more affected than men, but no difference was found among the different age groups. Prolonged disease progression was the most common AE, followed by deliberate self-harm and mental injury.

There are few retrospective record reviews based on a trigger tool approach with which to compare our results. Marcus et al. [10] investigated 8000 psychiatric hospitalisations in the United States and found a prevalence of 28% of patient safety events, including events with potentially negative consequences and adverse drug events, which were by far the most frequent (6%). Only 19% of AEs were considered preventable in this study, and almost 85% resulted in no or minor harm. In a recent publication from Singapore, 11% of the reviewed records had at least one mental healthcare-associated patient safety incident, and most of the events were aggressive behaviours [12]. In the same study, an AE of any kind was found in 19% of the patient records.

In comparison to somatic hospital healthcare with increasing frequencies of AEs with age [16], the equal distribution of AEs in different age groups is striking. In the present study, only 15% of the patients were 65 years or older. The corresponding figure for the Swedish somatic hospital healthcare was 66% [16]. One possible explanation for this difference in age distribution in somatic and psychiatric hospital care is that mental diseases usually have an earlier onset than somatic diseases. Another explanation is that older patients with concurrent mental and somatic diseases are usually cared for in somatic healthcare only and not captured in this study. A Swedish report from the National Board of Health and Welfare found that death was more common one year after admission in a somatic department for the treatment of depression, schizophrenia and abuse than if the treatment had been given in a department specialising in psychiatric care [24]. There were also less use of psychotropic drugs and fewer new admissions to in-hospital mental care after somatic physicians’ surveillance of the patients. This finding could indicate that psychiatric diseases were not taken care of in an optimal way in somatic departments. An epidemiological Danish study showed that psychiatric treatment in the preceding year was associated with an increased risk of dying from suicide [25], underlining the complexity of care for mental disorders.

Pharmacotherapy plays an important role in mental health treatment, and patients with serious psychiatric conditions can be vulnerable to risks and side effects associated with the treatment, such as cognitive impairment. This situation may affect the poor reporting of medication-related AEs. Different psychotropics can contribute to somatic health problems, such as impaired glucose tolerance and extrapyramidal side effects. Polypharmacy is common, thus increasing this risk. A systematic review [26] found that adverse drug events occurred in 10–42 per 1000 patient days in mental health hospitals, with psychotropic medication accounting for the majority of harmful events. In the present study, we found that 12% of the AEs were medication related. It is difficult to compare studies as several factors differ: health care systems; diagnostic panoramas; data sampling from trigger tools, incident reports, pharmacy or nurse staff reports; and data presentation. Still, our rate seem low and might be underreported. Approximately one-third of the medication-related AEs were considered avoidable. This result can be compared with the American Psychiatric Association’s action for patient safety [27], which estimated that half of drug-related AEs could have been prevented. Non-psychiatric drugs are associated with preventable AEs more often than psychiatric drugs [28, 29].

The majority of AEs as well as AEs causing severe or catastrophic harm affected women and for the latter group it was seen in all areas of AEs. This was a surprising finding and something that differs from what is known from somatic care [16]. Although sex differences have been observed across many psychiatric diseases [30], our cohort had equally distributed sex. We have not found other studies reporting on gender differences for AEs. In our study we did not investigate courses of harm. Our finding implies that preventive measures for women should be focused in clinical practice and further studies.

In our study, AEs were more common in hospital care than in out-patient care. This finding was expected, as care in hospitals is ongoing round the clock, patients are more severely ill and more advanced care is usually given. This condition contributes to an increased risk for AEs. However, the AE area ‘prolonged disease progression’ was the most common in out-patient care and more common than that in hospital care. This result can indicate low accessibility to and/or flexibility in out-patient care. It can also be explained by poor cooperation with the patient’s relatives. According to a recent report of the Swedish Health and Social Care Inspectorate (IVO), both patients and relatives have sparse influence on care planning [31]. Another difference between outpatient and hospital care becomes obvious when the incidence of attempted suicide is considered. Hospital care seems to provide the suicide preventative effect that is intended by hospitalisation. However, unfortunately, the same benefit is not seen in deliberate self-harm, the incidence of which is not reduced to the same extent.

As mental AEs affect young people of working age, the common AE ‘prolonged disease progression’ might have considerable economic consequences to society and to the affected individual.

The number of AEs in the area of ‘illegal/unethical treatment’ is very low in our study at 1%. Only 60% of these AEs were regarded as preventable. From a legal point of view, all AEs in this category should be preventable because they are against the law by definition. However, in an acute situation in which coercive measures were found necessary, the review teams might have chosen to consider the AE as not preventable.

The unexpectedly high frequency of triggers (78% of the records) indicating deficiency in the overall quality of care can have several explanations. The patient safety movement started in somatic healthcare and reached mental health services and psychiatric care at a later stage. In a national Swedish survey, the patient safety culture was rated lower than in somatic care [32]. The lack of documentation of physical status during psychiatric hospital care is troublesome, as patients with a serious mental illness have higher rates of physical conditions, such as obesity, diabetes, hypertension and HIV/AIDS [33, 34], and risk behaviour, such as smoking, drinking alcohol and physical inactivity [35].

Our study has several strengths. The review was undertaken on a national basis. No specific trigger tool was at hand, but there was profound experience in the research group from the development and national use of a trigger tool for somatic care. Our triggers and description of psychiatric AEs were formed after discussions in a national expert panel, patient organisations and round-table discussions. A preliminary version of the trigger tool was adjusted according to the testing results. The review teams were given centralised education, and support was available all throughout to synchronise the review process. This study is limited by the retrospective design with the risk of hindsight bias and the risk of information bias, as only what had been documented in the records could be assessed. No assessment of interrater reliability was conducted. It can sometimes be difficult to decide if a gradual worsening of a disease is due to deviation from standard care and thus regarded as an AE or the natural course in spite of correct treatment. Included causes for classification as AEs were for instance incomplete investigation, incorrect diagnosis or treatment inadequate or delayed leading to worsening of disease. Although thoroughly discussed during the second review stage, it remains a subjective decision.

## Conclusions

A trigger tool for the assessment of AEs in psychiatric care was developed and found useful. AEs seemed to be as common in mental as in somatic healthcare, but the majority of AEs resulted in minor or less harm. In contrast to somatic care, no differences were found in age in psychiatric care. This study provides further knowledge in the area of AEs in psychiatric care. As almost half of the identified AEs were assessed as preventable, there are clear indications for areas where efforts for improvement of patient safety could be intensified. Systematic interventions to improve the quality of psychiatric care are warranted.

## Data Availability

Data are available from the authors upon reasonable request and with permission of the Swedish Association of Local Authorities and Regions*.*

## Abbreviations

AE: Adverse event
CI: Confidence interval
IVO: Swedish Health and Social Care Inspectorate
